# Antioxidant Capacity as a Marker for Assessing the *In Vitro* Performance of the Endangered *Cistus heterophyllus*

**DOI:** 10.1155/2013/176295

**Published:** 2013-12-25

**Authors:** Antonio López-Orenes, Antonio F. Ros-Marín, María A. Ferrer, Antonio A. Calderón

**Affiliations:** Departamento de Ciencia y Tecnología Agraria, Universidad Politécnica de Cartagena, Paseo Alfonso XIII 48, 30203 Cartagena, Spain

## Abstract

*Cistus heterophyllus* subsp. *carthaginensis* is an endemic and endangered species from the SE Mediterranean coastal region of Spain. Within the framework of the efforts aiming to species conservation, *in vitro* culture techniques could be of interest. The aim of this study was to evaluate the antioxidant capacity of *C. heterophyllus* shoot cultures as a possible marker of *in vitro* performance. The effects of five different basal salt formulations and cytokinin levels on *in vitro* performance and antioxidant capacity were examined. K^+^/Na^+^ and Ca^2+^/Na^+^ ratios initially present in culture media greatly affected the antioxidant capacity (the lower the ratios the higher the antioxidant capacity). Increasing concentrations of BA resulted in higher antioxidant capacity. The results obtained point to antioxidant capacity as being a marker of incidence of stress conditions in *in vitro* cultured *C. heterophyllus*. A good correlation was found between antioxidant capacity and total soluble phenolics present in *Cistus* extracts. Catechin was identified in all the extracts and its levels were found to change parallel to the antioxidant capacity, pointing to a prominent role played by this flavonoid in *C. heterophyllus* defence against oxidative stress, which in turn affects the *in vitro* performance of this species.

## 1. Introduction

The genus *Cistus* comprises around 20 species with a predominantly Mediterranean distribution [1]. Cistaceae species are main components of the shrublands in this area and they are also prominent members of the understory vegetation beneath Mediterranean forests. These species seem to be essential for the development of some processes taking place in several Mediterranean ecosystems and hence the importance of preserving them ([2] and references herein). Furthermore, many species of this botanical family, if not all of them, have been used since ancient times in folk medicine ([3] and references herein) and as ornamental plants, which encourages the interest for these plants and their conservation.

*Cistus heterophyllus* subsp. *carthaginensis* is a beautiful endangered plant whose European population is exclusively located in the provinces of Valencia and Murcia, in eastern Spain. Valencia population is reduced to only three spontaneously grown plants, being the Murcia population composed of no more than two dozen pure specimens associated to a sparse *Pinus halepensis* copse. These scant populations are severely threatened by several factors including low propagation and germination rates, hybridization with *C. albidus*, deforestation, and soil contamination due to wastes generated from extraction and processing of mineral resources (mainly Pb- and Zn-containing residues). As a result, this species is included in the critically endangered category (CR) of the red list guidelines by the Flora Commission of the Spanish Committee of the International Union for Conservation of Nature (IUCN) [4].

*In vitro* multiplication techniques have often been successfully utilized in propagation of rare and endangered species of plants, including some belonging to the genus *Cistus* [5], and therefore can contribute to the protection of plant genetic resources. However, the introduction and proliferation of explants *in vitro* can promote the formation of reactive oxygen species (ROS) due to, among other factors, wounding, mechanical perturbations, high osmoticity, abnormal mineral nutrition, and unusual hormonal treatment [6]. ROS play an important signaling role in plants [7] although at high concentrations they can cause oxidative stress [6, 8]. Oxidative stress defines the consequences of a mismatch between the production of ROS and the ability to defend against them. It has been demonstrated that oxidative stress contributes to recalcitrance [9], provokes several plant tissue culture physiological disorders such as hyperhydricity [6, 8, 10], and affects the development and the morphogenic responses of tissues grown *in vitro* [11].

Plants combat oxidative damage by a broad spectrum of ROS-scavenger systems, including antioxidative enzymes as well as nonenzymatic molecules such as ascorbate, glutathione, carotenoids, *α*-tocopherol, and phenolic compounds. In addition to a crucial role in cellular ROS homeostasis, this large and diverse group of antioxidants influences plant growth and development by modulating processes such as mitosis and cell elongation [7, 12].

A relatively high level of antioxidants in plant tissues could be considered to be an indicator of incidence of stress conditions and could be crucial for plants to tolerate oxidative stress and, hence, many environmental unfavourable conditions ([13] and references cited herein). Since *in vitro* culture offers the possibility to easily change medium composition, this methodology can be considered of great interest in order to modulate the performance of plant tissues [14]. Concentration of mineral nutrients, vitamins, and phytoregulators levels even physical state of culture media can influence growth and multiplication rate, as well as accumulation of phytochemicals in plant vitro cultures [15–18] which, in turn, could be essential for plant survival upon transfer to field conditions.

The main goal of this work was to study how the medium composition affects both the performance and the antioxidant capacity of *C. heterophyllus* shoots cultured *in vitro*. Presence or absence of agar, basal salt formulation, and BA concentration was evaluated for shoot proliferation rates, photosynthetic pigment content, and antioxidant capacity in methanolic extracts. Since pioneering work on this species by Arregui et al. [19] and to the best of our knowledge, no other report studying the effect of basic constituents of culture medium on *in vitro* performance of *C. heterophyllus *has been published. In comparison to that work what the present one provides is a more extensive study on the effect of medium components and their impact on shoot antioxidant capacity, the latter probably being crucial for obtaining more vigorous plants and, in turn, plant material more appropriate for their reintroduction in the framework of *Cistus* recovering programs.

## 2. Materials and Methods

### 2.1. Plant Material

**Figure 1:**
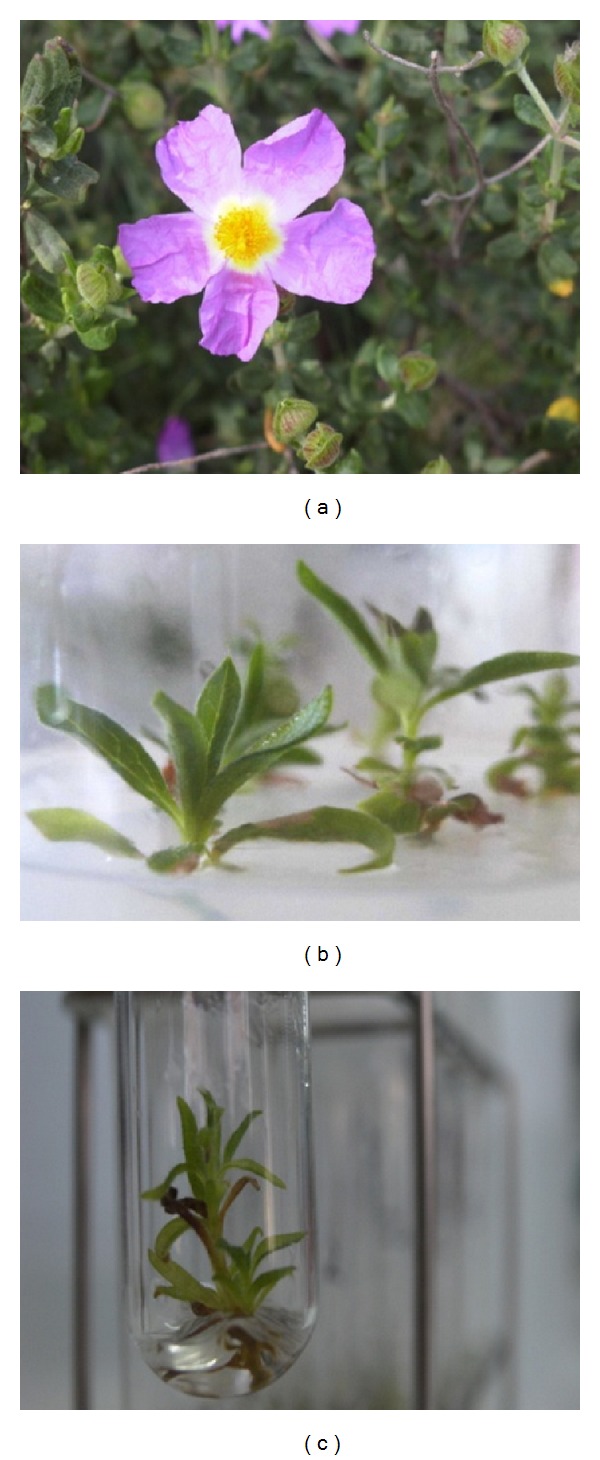
Micropropagation of *Cistus heterophyllus *from an adult plant. (a) Detail of a mature field-grown plant in June. Shoots developed on gelled-MS/2 (b) and liquid MS/2 media (c).

Stem cuttings of *C. heterophyllus* were obtained from a mature old plant growing in the Sierra de Cartagena (Figure 1(a)). The stems were washed in running water and then surface-sterilized with 70% (v/v) ethanol for 25 seconds and 10% Domestos hypochlorite solution for 20 min. They were then rinsed thoroughly three times with sterile distilled water. Subsequently, 2 cm long nodal explants bearing two axillary buds were excised and cultured. The material was maintained by monthly subculture as *in vitro* shoots on MS medium [20] without growth regulators and solidified with 0.8% Difco Bacto agar. All cultures were kept at 25°C in a 14 h photoperiod (12 W m^−2^; cool-white fluorescent lights Philips F40 CW).

### 2.2. Shoot Multiplication

Five-week-old shoots obtained on establishment medium were selected for multiplication experiments. Nodal segments (1 cm long) each containing a pair of axillary buds were cultured on the following five combinations of macro- and micronutrients: MS [20], MS/2 (MS with macronutrients at half-strength), G [21], W [22], and DKW [23]. All media were supplemented with casein hydrolysate (250 mg L^−1^), sucrose (3%, w/v), and MS vitamins [20]. The pH was adjusted to 5.8 before adding or not 0.8% (w/v) Difco Bacto agar. Aliquots of solid (15 mL) and liquid (250 *μ*L) medium were dispensed into jar or test tubes, respectively, and autoclaved at 121°C and 1.1 kg cm^−2^ for 20 min. All treatments carried out (see below) were assessed 4 weeks after inoculation.

In order to determine the effects of 6-benzylaminopurine (BA) on culture performance, MS/2 liquid media were supplemented with the cytokinin at various concentrations (0.0, 0.1, 1.0, and 5.0 mg L^−1^). Samples for subsequent analyses were taken after 1 or 4 weeks of culture.

### 2.3. Chlorophylls and Carotenoids Determinations

Shoot explants were ground with a mortar and pestle in the presence of 80% (v/v) acetone. After centrifugation of the extracts for 10 min at 10,000 g, supernatants O.D. was measured at 470, 646, and 663 nm using a Shimadzu UV-1603 spectrophotometer. The extinction coefficients and the equations reported by Lichtenthaler and Wellburn [24] were used to calculate the amounts of total carotenoids and chlorophylls *a* and *b*.

### 2.4. Extraction and Quantification of Total Soluble Phenols

The phenolic compounds from shoot tissues were extracted by homogenizing the samples in 70% (v/v) methanol, using a mortar and pestle. The extracts were maintained in an ice bath for 1 h and centrifuged at 3,000 g for 40 min at 4°C. Distilled water was added to the supernatants to lower the methanol concentration to 3% (v/v). The diluted extracts were then loaded onto Sep-Pak C18 cartridges (Waters Corporation). Finally, after washing the cartridges with water, phenolics were eluted with 1.5 mL of methanol. One hundred microlitres of these fractions was used for the determination of total soluble phenolic compounds using the Folin-Ciocalteu reagent [25]. Caffeic acid was used as standard for quantification purposes.

### 2.5. HPLC Analysis

RP-HPLC (reversed phase-high pressure liquid chromatography) assays were performed with a liquid chromatographic system equipped with a Waters Alliance 2695 separations module (Waters, Milford, MA, USA), a variable-wavelength diode array detector Waters 2996, and controlled by Millennium 32 software. A Luna C-18 reversed-phase column (250 mm × 4 mm, 5 *μ*m particle size) supplied by Phenomenex was employed. Separation was carried out at room temperature as described by Hernández et al. [26]. The flow rate was 0.8 mL min^−1^, and the injection volume was 20 *μ*L. All solutions and HPLC mobile phases were prepared with freshly MilliQ water and filtered through 0.45 *μ*m nylon filters (Millipore, Bedford, MA, USA).

The presence of catechin was confirmed further by HPLC-ESI-MS (Waters Alliance 2695 separations module coupled to Waters ZQ 4000 detector) by applying a declustering potential of −60 V. Chromatographic conditions were the same previously described [26].

### 2.6. Antioxidant Capacity Assessed by DPPH Radical Scavenging

Antioxidant potential was assessed according to Brand-Williams et al. [27] with minor modifications. Briefly, fifty microlitres of a series of diluted methanolic extracts obtained as described above was added to 1 mL of a 0.004% methanol solution of DPPH. Absorbance at 515 nm was determined after a 25 min incubation period at room temperature in the dark. In order to compare the results obtained in the different treatments, the radical scavenging activity was calculated using the following equation: [(Ac − As)/Ac] × (1/g FW) (where Ac = absorbance of the DPPH solution + 50 *μ*L methanol, As = absorbance of reaction media in the presence of sample, and g FW = fresh weight of tissue in grams).

### 2.7. Data Analysis

Explants were randomly assigned to treatments, and data were recorded as means ± standard errors (SE) of 4–6 replicates per treatment in three repeated experiments. Statistical analyses were performed using the software provided by Sigmaplot Jandel Scientific and the SPSS software package (version 19.0; SPSS Inc., Chicago, IL, USA).

## 3. Results and Discussion

### 3.1. Influence of Medium Solidification and Macro- and Micronutrients Composition on Shoot Multiplication

Table 1 shows the effect of different mineral nutrient compositions on the multiplication rate of *C. heterophyllus *explants cultured on both solid and liquid media. As can be observed, nodal segments were capable of directly developing several nodes on all basal media tested in the absence of growth regulators. However, a considerable decline in the number of nodal segments produced per explant was observed in both agar-solidified and liquid W media. Growth and morphogenesis of plant tissues under *in vitro* conditions are largely influenced by both qualitative and quantitative composition of culture media [14]. Presumably, the low levels of mineral nutrients present in W medium might contribute to the decrease of shoot growth rate. In fact, total concentration of salts constituting W medium is around 7.5 mM, whereas in the other media assayed this amount ranges between approximately 23 mM (MS/2) and 46 mM (MS). So, a rapid depletion of essential nutrients in cultures containing W medium would be expected and, hence, an early cessation of shoot growth.

Apparently, liquid media are superior to solid media as regards shoot multiplication rate. As can be observed in Table 1, a 4-week cultivation period in liquid media gave higher proliferation rates compared with that obtained on solid media, irrespective of the basal formulation assayed. The greater water availability for cellular expansion caused by the increase of water potential in the liquid media could explain this effect. However, excessive water availability could lead to the loss of control of water uptake in tissues and may originate the physiological disorder called hyperhydricity [28].

No hyperhydricity symptoms were observed in the assays carried out with *C. heterophyllus* (Figure 1). Figure 1(b) illustrates the healthful appearance of the new shoots developed in MS/2 basal liquid medium. However, in order to ascertain that an excessive water accumulation was not taking place, tissue water content (TWC) and biomass accumulation were analyzed (Table 2). Compared with gelled MS/2 medium, no differences in TWC among shoots developed in liquid basal media were observed. The biomass accumulation was also similar in all the liquid media tested, with the exception of W medium which presented the lowest dry weight (Table 2). As mentioned above, the low concentration of inorganic nutrients on W medium, especially N and P, may have contributed to the reduction in shoot growth.

### 3.2. Influence of Macro- and Micronutrient Compositions on Shoot Quality and Antioxidant Capacity in Liquid Media

The content and relationship among photosynthetic pigments could be used to assess quality of *in vitro* propagated material since they represent a measure of photosynthetic activity of shoots. A higher photosynthetic capacity *in vitro* should result in more rapid growth and better plantlet acclimatization and survival success during the transition from *in vitro* to *ex vitro* environment [29]. Chlorophyll content in *C. heterophyllus* shoots was not significantly changed by the composition of the media formulation used, with the exception of DKW medium in which the chlorophyll levels found were slightly smaller (Table 3). All the shoots showed a Chl a/b ratio in the range of 2.0–2.5 with the exception of shoots grown in MS where the Chl a/b ratio increased to 3.4. As regards the carotenoid content, shoots developed in G showed lower pigment levels than those found in shoots grown in the other media assayed (Table 3).

Photosynthetic pigment composition can be considered as a good indicator of vitality in higher plants. In our study, levels of both chlorophylls and carotenoids in shoots were relatively low irrespective of the medium formulation used. These results are in accordance with many previous studies and could be the consequence of cultural conditions during *in vitro* growth. In this way, high air humidity, low irradiance, low air exchange, and presence of high sugar concentrations as carbon source probably limit pigment accumulation and hence photosynthetic capacity [30]. Although there were not significant differences in chlorophyll levels among shoots cultured in the media assayed, the lower Chl a/b ratio found for shoots grown in W could be the consequence of a highly disproportionate amount of reduced carbon over mineral nutrients (mainly N). As Chl a/b ratio has been described to be positively correlated with the ratio of Photosystem II-cores (PSII) to light harvesting chlorophyll-protein complex (LHC II) [31], a decrease in this parameter could indicate an increase in energy dissipation through nonphotochemical processes, suggesting the expression of a robust antioxidant system in chloroplasts of *Cistus* shoots cultured in W.

An integrated parameter to evaluate the full spectrum of antioxidant compounds present in a tissue is the total antioxidant capacity [32]. Among the methods available for the measurement of total antioxidant capacity, the DPPH (1,1-Diphenyl-2-picrylhydrazyl radical) assay is widely employed because of simplicity and high reproducibility. This spectrophotometric method employs a stable nitrogen-centered free radical (DPPH^●^) in a methanol solution. In its radical form, DPPH^●^ has an absorption band at 515 nm which disappears upon reduction. Molecules which are able to perform this reaction can be considered as radical scavengers and therefore antioxidants [27].

**Figure 2:**
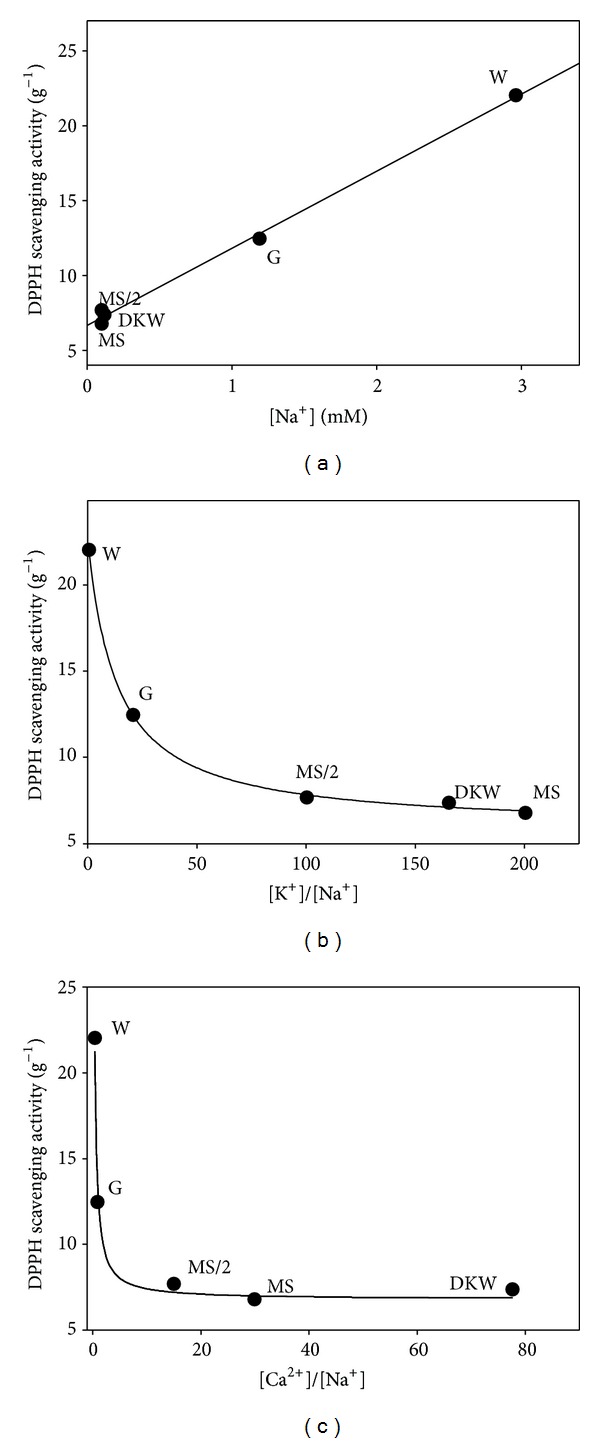
(a) Relationship between initial Na^+^ concentrations present in the basal media formulations used in this study and antioxidant activity displayed by* C. heterophyllus* extracts after 4 weeks of culture in liquid media. In (b) and (c) DPPH scavenging activity versus K^+^/Na^+^ and Ca^2+^/Na^+^ ratios, respectively, is shown.

Table 4 shows the effect of macro- and micronutrient formulations on the free radical scavenging capacity of *Cistus* shoots grown in liquid media. Surprisingly, shoots grown in W medium exhibited the highest antioxidant activity, followed by those developed in G medium. Although W medium presents low amount of some macronutrients, like phosphorus (0.14 mM), nitrate (0.79 mM), and potassium (1.69 mM), there were no significant trends between DPPH radical scavenging capacity and the levels of these macroelements present in the basal media when regression analyses were carried out (data not shown). However, a significant trend (*r* ^2^ = 0.9967) was found between DPPH radical scavenging capacity and Na^+^ levels present in the basal media (Figure 2(a)). It is well established that external Na^+^ negatively impacts intracellular K^+^ influx, attenuating acquisition of this essential nutrient by cells [33]. As can also be observed in Figure 2(b), the curve representing the antioxidant capacity versus K^+^/Na^+^ ratio fitted well with a rational function with an asymptotic horizontal value calculated to be 5.85 g^−1^ (which could be considered as the antioxidant “constitutive” level in *C. heterophyllus* shoot cultures). Data in Figure 2(b) also indicates that K^+^/Na^+^ ratios below 50–60 (in a molar basis) enhanced the production of antioxidants, probably as a consequence of the incidence of oxidative stress conditions. Moreover, a similar curve was observed when antioxidant activities were represented versus Ca^2+^/Na^+^ ratios in the shoot incubation media (Figure 2(c)). These two indexes, K^+^/Na^+^ and Ca^2+^/Na^+^ ratios in plant tissues, have been suggested as markers of Na^+^-related salt sensitivity [34] and in two species of the genus *Cistus* (*C. albidus* and *C. monspeliensis*) a negative correlation between Na^+^ content and salt tolerance has been reported [35]. All these facts point to the possibility that an imbalance in the K^+^/Na^+^ and Ca^2+^/Na^+^ ratios provoked the impairment of redox status and, as a consequence, a rise in antioxidant capacity of *C. heterophyllus* shoots. The relationship among K^+^/Na^+^ cellular homeostasis, hydrogen peroxide and nitric oxide levels, and the activities of antioxidant enzymes has previously been reported in two poplar cell lines differing in salt tolerance [36]. From that work it can be concluded that the stress signals (H_2_O_2_ and NO) generated by the tolerant poplar species in response to NaCl are responsible for the activation of both ion transporters and cellular antioxidant systems, which in turn contribute to the expression of salt tolerance [36].

In the present work, *Cistus* explants under less-favourable conditions were able to increase the levels of antioxidant metabolites, which could contribute to reduce the stress generated in these circumstances. The observed high levels of DPPH radical scavenging capacity could reflect the expression of antioxidant biosynthetic pathways resulting in prevention or minimization of the cytotoxic impact of ROS formed during stress conditions, as it has also been described in other plant materials for increased tolerance against chilling [37] and salt stress [38], among other abiotic stressing factors.

### 3.3. Effect of BA Treatments on the Antioxidant Properties of *Cistus* Shoots

**Figure 3:**
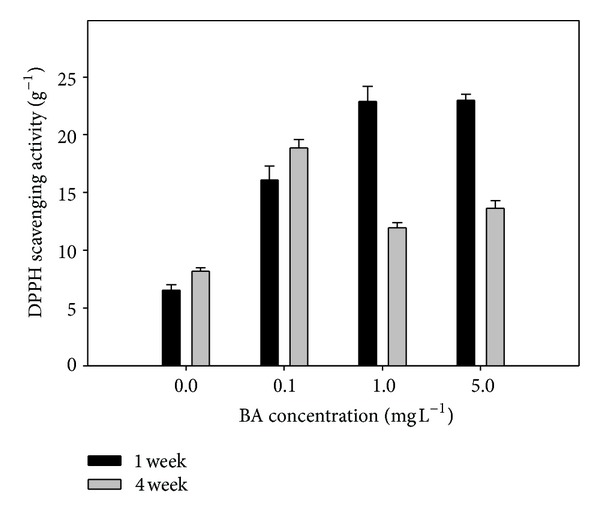
Effect of BA concentration on antioxidant capacity of *C. heterophyllus* shoots after 1 or 4 weeks of culture in MS/2 liquid media. Vertical bars represent means ± standard error.

**Figure 4:**
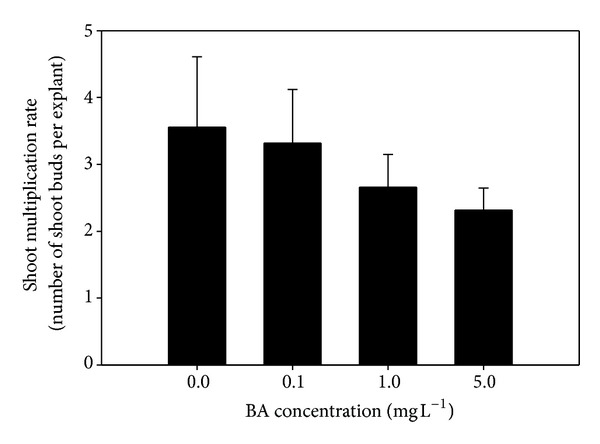
Effect of BA concentration on *C. heterophyllus* shoot multiplication rate after 4 weeks of culture in MS/2 liquid media supplemented with 0.0, 0.1, 1.0, and 5.0 mg L^−1^ BA. Vertical bars represent means ± standard error.

Cytokinins are common components of plant tissue culture media, especially those designed for regeneration and micropropagation, and this is the reason why there are many studies on the effects of these phytoregulators on plant proliferation.  Figure 3 shows the effect of BA on the antioxidant properties of *Cistus* shoots grown in MS/2 liquid media. From this figure it can be observed that the addition of BA provoked a significant, dose-dependent increase in the free radical scavenging capacity of *Cistus* extracts after a 1-week incubation period. However, as can also be seen in Figure 3, DPPH scavenging activities decreased for the higher BA concentrations after 4 weeks. These results seem to point out that BA stimulates the production of antioxidant compounds in *C. heterophyllus* liquid shoot cultures at short term incubations, probably due to the stress originated by the addition to culture media of the phytoregulator. However, at longer times, persistence of high levels of the stressor could lead to the onset of deteriorative processes in shoot cultures. Additional evidence for this statement arose from chlorophyll analysis where ratio Chl a/b decreased from 2.57 (control) to 1.45 (BA 5 mg L^−1^) after the 4-week incubation period (data not shown). Data on shoot multiplication rate support this view. So, increasing concentrations of BA in MS/2 liquid basal media tended to decrease *Cistus* proliferation rate (Figure 4).

In this way, it has been reported that cytokinins, especially BA, at high doses, can act as programmed cell death inducers in plants [39]. This apparently paradoxical prosenescence effect of BA could be initiated by the phytoregulator phosphorylation and probably course through ATP depletion and ROS generation [40]. What is more, transgenic plants overproducing cytokinins exhibit typical traits of plants being submitted to stress conditions [41, 42], including overexpression of antioxidant enzyme systems [43]. Survival of plant material in the presence of high doses of cytokinins would depend on the appropriate expression (in both time and intensity) of cell defence systems in order to prevent or palliate cytokinin-induced damages.

Several authors have linked cytokinin contents (or treatments) to plant tolerance against both abiotic and biotic stress agents [44, 45]. Increased production of cytokinins or exogenous application of these phytoregulators has been reported not only to increase tolerance to mild stress but also to speed up plant recovery after cessation of stress conditions (see [44] and references cited herein). Proposed mechanisms for these effects include the above mentioned upregulation of antioxidant enzyme activities [43], as well as a direct effect of cytokinins acting as ROS scavengers [46, 47].

**Figure 5:**
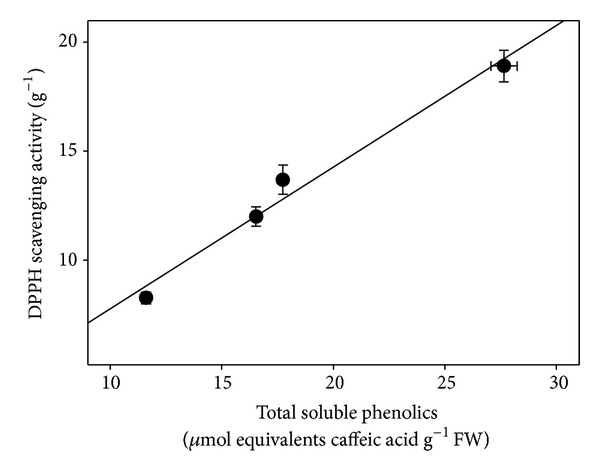
Relationship between DPPH scavenging activity and total soluble phenolic contents in *C. heterophyllus* shoots cultured for 4 weeks in MS/2 liquid media supplemented with different BA concentrations. Values represent means ± standard error.

Increased accumulation of phenolic compounds in transgenic tobacco plants overexpressing cytokinin biosynthetic genes has been described and related to the stress response provoked by high hormone levels [48]. Many phenolics can act as antioxidants due to their high reactivity as hydrogen or electron donors, the ability of their radical forms to stabilize and delocalize unpaired electrons, and their ability to chelate transition metal ions [49]. In the present study phenolic compounds could be responsible for a great part of the antioxidant activity found in *C. heterophyllus* extracts. In fact, a good correlation (*r* ^2^ = 0.9795) between soluble phenol contents present in BA-treated* Cistus* shoots and free radical scavenging capacity was found (Figure 5). Although there is relatively little information concerning the phenolic composition of this species, different flavonoids, especially flavan-3-ols, have been reported to occur in other members of the genus *Cistus* [50] and it is widely accepted that these compounds possess high antioxidant and antiradical properties.

**Figure 6:**
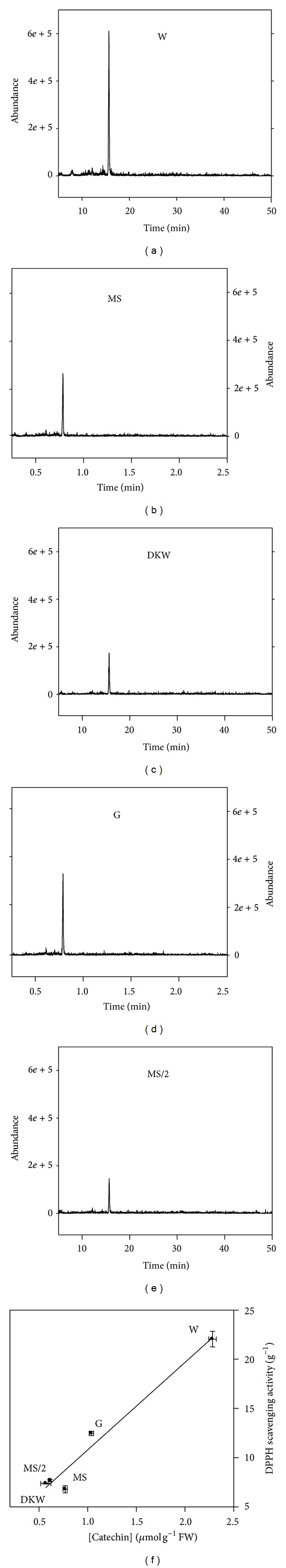
(a)–(e) Selected negative-ion HPLC-ESI-MS chromatograms of *m/z* = 289 corresponding to extracts of *C. heterophyllus* shoots cultured for 4 weeks in liquid medium containing the different basal media formulations used in this study. (f) Relationship between DPPH scavenging activity and catechin contents in shoots cultured for 4 weeks in liquid media containing the following basal media: G, Gamborg's B5; DKW, DKW/Juglans; MS, full strength Murashige and Skoog; MS/2, half-strength macronutrients Murashige and Skoog; W, White.

Identification and quantification by HPLC-DAD-MS of individual phenolics present in *C*.* heterophyllus* shoot cultures showed that the flavan-3-ol (+)-catechin was present in all the extracts analyzed. Figures 6(a)–6(d) show the occurrence of the characteristic fragment ion corresponding to catechin (*m/z* 289, [M–H]^−^) in the extracts obtained from shoots grown in the five different basal media assayed. Furthermore, a good correlation was found between the catechin content of the extracts and the antioxidant capacity determined by the DPPH method (Figure 6(e)), suggesting a prominent role for catechin in the response of *C. heterophyllus* to stressful conditions. In this way, preliminary results on the effect of heavy metal on *Cistus* shoots cultured *in vitro* have also shown a noticeable increase in catechin levels (manuscript in preparation) supporting the view that this compound is a key element in the antioxidative response of the species. *C. heterophyllus* shoot cultures treated with increasing concentrations of salicylic acid have recently been reported to contain increased levels of flavan-3-ols, although, in this case, a decrease in soluble flavanols was related to the rise in proanthocyanidins and to the reduction in shoot growth for the highest concentrations of the phytohormone [51]. The involvement of flavan-3-ols in coping with stress conditions has also been reported in other members of the genus *Cistus*. So, Hernández et al. [52] showed the occurrence of enhanced levels of monomeric and polymeric flavanols in *C. clusii* submitted to excess light stress conditions or as a consequence of plant ageing.

Despite the fact that flavonoids and other compounds with demonstrated *in vitro* antioxidant activity have been reported to be accumulated in both abiotic and biotic stress conditions, their exact role in alleviation of the oxidative stress that follows a stress agent challenge is still controversial [53]. Flavonoids could exert their protective role in both a direct and an indirect way. The direct effect implies that the antioxidant compound comes in contact with ROS, or any other pro-oxidant agent, while the indirect action can involve several reactions such as metal chelation and radiation screening [53]. Another interesting indirect mechanism of protection against oxidative stress is based on the use of secondary metabolism biosynthetic pathways to channel photoassimilates, this constituting an alternative route for photochemical energy dissipation and hence for avoiding the overenergization of the photosynthetic apparatus [54]. Operation of the latter mechanism brings as a consequence the withdrawal of constituent elements necessary for plant growth, resulting in the poor performance of plant material cultured under soft or mild stress conditions.

In conclusion, this study has shown that the performance of *Cistus heterophyllus* shoots cultured *in vitro* is considerably modulated by both the physical state and the chemical composition of culture media. Ionic balances (particularly K^+^/Na^+^ and Ca^2+^/Na^+^ ratios) and BA concentration in culture media dramatically affected the antioxidant capacity of *Cistus* shoots, phenolic compound levels being well correlated with antioxidant activity in plant material. These observations suggest a major role for these compounds, especially for catechin, in the *C. heterophyllus* defence response against oxidative stress. Since phenolics are also involved in key developmental processes, the selection of proper media formulations plays a crucial role in establishing both an efficient tissue culture system for plant propagation and the foundations for profitable secondary metabolite production. From a conservational point of view, *in vitro* culture techniques appear to be powerful tools to ensure *C. heterophyllus* survival. The improvement of *in vitro* plant performance is crucial to accomplish multiplication and reintroduction in its natural habitat of this critically endangered species.

## Figures and Tables

**Table 1:** Effect of basal media formulations on shoot multiplication of *Cistus heterophyllus* after 4 weeks of culture on agar-solidified media and liquid media.

Basal medium	Shoot multiplication rate (mean number of newly formed nodes per explant)	
	Solid medium	Liquid medium
DKW	2.20 ± 0.50^a^	3.14 ± 0.40^a^
G	2.00 ± 0.45^a^	3.25 ± 0.31^a^
MS	1.60 ± 0.40^a^	3.00 ± 0.44^a^
MS/2	2.37 ± 0.89^a^	3.57 ± 1.05^a^
W	1.00 ± 0.00^b^	1.43 ± 0.30^b^

^a,b^Values are means ± SE. Means followed by the same letter were not significantly different according to Tukey's HSD test (*P* < 0.05).

**Table 2:** Effect of basal media formulations on tissue water content (TWC) and biomass accumulation in shoots of *C. heterophyllus* after 4 weeks of culture in liquid media.

Basal medium	Tissue water content (%)	Dry weight (mg/shoot)
DKW	94.1 ± 0.5^a^	7.1 ± 0.9^a^
G	91.8 ± 0.8^b^	7.5 ± 0.9^a^
MS	92.4 ± 1.9^a, b^	6.0 ± 2.3^a^
MS/2		
Solid	89.7 ± 0.3^b^	10.0 ± 2.0^a^
Liquid	91.1 ± 1.4^b^	6.4 ± 1.0^a^
W	91.7 ± 0.6^b^	4.0 ± 1.4^b^

^a,b^Values are means ± SE. Means followed by the same letter were not significantly different according to Tukey's HSD test (*P* ≤ 0.05).

**Table 3:** Effect of basal media formulations on total chlorophyll (chl *a* + *b*) content, chl *a*/*b* ratio, and carotenoids levels of *C. heterophyllus* after 4 weeks of culture in liquid media.

Basal medium	Total Chl (*μ*g g^−1^ FW)	Chl *a/b* ratio	Carotenoids (*μ*g g^−1^ FW)
DKW	378 ± 65^b^	2.53 ± 0.01	44.71 ± 3.12^a^
G	425 ± 17^b^	2.52 ± 0.51	24.40 ± 5.05^b^
MS	585 ± 71^a^	3.41 ± 0.31	53.59 ± 9.31^a^
MS/2	556 ± 81^a^	2.57 ± 0.31	48.38 ± 3.55^a^
W	554 ± 86^a^	2.06 ± 0.01	38.75 ± 6.65^a^

^a,b^Values are means ± SE. Means followed by the same letter were not significantly different according to Tukey's HSD test (*P* ≤ 0.05).

**Table 4:** Effect of basal media formulations on DPPH radical scavenging capacity of *C. heterophyllus* after 4 weeks of culture in liquid media.

Basal medium	DPPH radical scavenging capacity (g^−1^)
DKW	7.36 ± 0.06^c^
G	12.45 ± 0.22^b^
MS	6.77 ± 0.36^c^
MS/2	7.67 ± 0.10^c^
W	22.03 ± 0.79^a^

^a,b,c^Values are means ± SE. Means followed by the same letter were not significantly different according to Tukey's HSD test (*P* < 0.05).

## Abbreviations

BA: N^6^-benzyladenine
DKW: DKW/Juglans medium
DPPH: 1,1-Diphenyl-2-picrylhydrazyl radical
HPLC-ESI-MS: High performance liquid chromatography coupled to electrospray ionization mass spectrometry
MS: Full strength Murashige and Skoog medium
MS/2: Half-strength (half macronutrients) Murashige and Skoog medium
G: Gamborg's B5 medium
ROS: Reactive oxygen species
W: White medium.
